# Expression of antiapoptosis gene survivin in luteinized ovarian granulosa cells of women undergoing IVF or ICSI and embryo transfer: clinical correlations

**DOI:** 10.1186/1477-7827-10-74

**Published:** 2012-09-07

**Authors:** Michail Varras, Katerina Polonifi, Marina Mantzourani, Konstantinos Stefanidis, Zacharias Papadopoulos, Christodoulos Akrivis, Aris Antsaklis

**Affiliations:** 1 First University Department of Obstetrics and Gynecology, “Alexandra” General Hospital, University of Athens, Athens, Greece; 2 Third Department of Obstetrics and Gynecology, “Elena Venizelou” General Maternity Hospital, Athens, Greece; 3 First University Department of Internal Medicine, University of Athens, Laiko General Hospital, Athens, Greece; 4 Department of Obstetrics and Gynecology, “G Chatzikosta” General State Hospital, Ioannina, Greece

**Keywords:** Granulosa cells, Infertile patients, Survivin, IVF, ICSI

## Abstract

**Background:**

The purpose of the study was to determine the incidence of survivin gene expression in human granulosa cells during ovarian stimulation in Greek women with normal FSH levels, undergoing IVF or ICSI and to discover any correlation between levels of gene expression and clinical parameters, efficacy of ovulation or outcomes of assisted reproduction.

**Methods:**

Twenty nine women underwent ovulation induction for IVF or ICSI and ET with standard GnRH analogue-recombinant FSH protocol. Infertility causes were male and tubal factor. Cumulus–mature oocyte complexes were denuded and the granulosa cells were analyzed for each patient separately using quantitative reverse transcription polymerase chain reaction analysis for survivin gene expression with internal standard the ABL gene.

**Results:**

The ABL and survivin mRNA were detected in granulosa cells in 93.1%. The expression levels of survivin were significantly lower in normal women (male infertility factor) compared to women with tubal infertility factor (p = 0.007). There was no additional statistically significant correlation between levels of survivin expression and estradiol levels or dosage of FSH for ovulation induction or number of dominant follicles aspirated or number of retrieved oocytes or embryo grade or clinical pregnancy rates respectively.

**Conclusions:**

High levels of survivin mRNA expression in luteinized granulosa cells in cases with tubal infertility seem to protect ovaries from follicular apoptosis. A subpopulation of patients with low levels of survivin mRNA in granulosa cells might benefit with ICSI treatment to bypass possible natural barriers of sperm-oocyte interactions.

## Background

Cells originating from the follicular *stratum granulosum* layer differentiate into two types: the cumulus granulosa cells, which are in physical contact with the developing oocyte via cytoplasmic projections, and are associated with the oocyte development and the mural granulosa cells, which are situated on the theca interna and are essential for oestrogen production and follicular rupture [1,2]. In the adult human ovary, there are paracrine communications between the oocyte and granulosa cells throughout the growth and development of the oocyte and the ovarian follicle. Oocytes from primordial follicles fail to grow *in vitro* in the absence of granulosa cells. Cumulus granulosa cells play an important role in regulating oocyte maturation and the oocyte is also an important modulator of cumulus granulosa cell function [3]. In the field of assisted reproduction, the selection of embryos with high implantation potential remains one of the major goals in order to transfer one embryo and avoid therefore the adverse outcomes related to multiple pregnancies [4]. Studies of gene expression in cumulus granulosa cells in assisted reproduction treatment (ART) cycles are attractive non-invasive approaches to evaluate the fertility potential of the individual oocyte without compromising the oocyte integrity because any abnormal patterns of gene expression in cumulus cells could be either causes or consequences of abnormal development of the oocyte [3]. In addition, the studies in humans do not allow direct access to oocytes, while granulosa cells are easily available since they are always discarded before ICSI procedures [4].

Apoptosis is the cellular mechanism involved in the ovarian follicular atresia and luteolysis [5,6]. In the early stage of follicular development (primordial, primary, and small preantral), atresia is initiated by oocyte apoptosis followed by death of the granulosa cells [6,7]. Atresia of maturing (late preantral, antral) and mature (subordinate preovulatory) follicles is first demarcated by scattered granulosa cell apoptosis. As atresia progresses in these follicles, the number of dying granulosa cells increases dramatically, and large masses of apoptotic bodies are shed into the antral space [6,8]. Gonadotropins and estrogens prevent granulosa cells apoptosis and stimulate their mitotic activity, whereas androgens enhance granulosa cells death by apoptosis [9]. In addition, it has been found that lanosterol metabolic products regulate apoptosis during follicular development in mice [10]. Apoptosis of granulosa cells seems to have a negative effect on IVF outcomes [11-13]. A higher incidence of apoptotic granulosa cells has been associated with a higher incidence of empty follicles, fewer oocytes retrieved, empty follicles, poor quality of oocytes and embryos development and poor fertilization [11,14]. Furthermore, different ovarian stimulation protocols affect the incidence of apoptotic granulosa cells differently [15]. Inhibitor of apoptosis (IAP) proteins can inhibit the downstream components of the caspase activation pathways in the regulation of apoptosis and play important roles in regulating the progress of apoptosis [16]. A member of the IAP gene family is survivin which is located on chromosome 17q25 and encodes a protein, which is comprised of 142 amino acids, has a molecular weight of approximately 16.5 kD and is divided into four exons and three introns. Alternative splicing of the survivin gene transcript produces a number of different survivin splice variant mRNAs, which encode different proteins [17]. Survivin participates in the regulation of apoptosis by direct or indirect inhibition of the effector cell death proteases caspase-3 and caspase-7 and regulates the cell cycle in the G_2_/M phase by associating with mitotic spindle microtubules [18,19]. Survivin is abundantly expressed during fetal development, but is down-regulated in most adult tissues [19-24], except for expression in normal tissues such as skin, thymus, endothelial cells, proliferative and secretory endometrium and granulosa cells [20,25-30]. Survivin is, however, expressed in a wide variety of human cancers, including stomach, colorectal, lung, breast, pancreatic, prostate, and non-Hodgkin lymphoma in a prevalence between 30% and 100% [17,19-22,25,31-33]. When analyzed retrospectively, cancer patients with tumors expressing survivin exhibited shortened survival, association with unfavorable markers of disease progression, accelerated rates of recurrence, and increased resistance to therapy [20,21]. Johnson et al., (2002) indicated that survivin could play an important role in granulosa cells, acting as a functional protein associated with regulation of the cell cycle and the inhibition of apoptosis [29]. In addition, Fujino et al., (2008) studied the expression of survivin in infertile patients and found that the gene expression levels of survivin in patients with endometriosis were significantly lower than those in patients with male factor infertility. The gene expression levels of survivin in total pregnant patients were higher than those in total nonpregnant patients and than those in the male factor infertility [34].

At present, there are no morphological or physiological features of oocytes that can predict whether IVF fertilization will be successful, or whether is a need for ICSI [35]. Recently, the use of ICSI in cases unrelated to male factor infertility has increased greatly at ART facilities [35-37]. Such general use of ICSI, however, raises concerns because intracytoplasmic sperm injection is more expensive and time-consuming technique that requires special equipment and skills. In addition, an increase in the risk of transmitting chromosomal anomalies or imprinting disorders has been described [38-40], although it is not clear whether these risks are due to the procedure or to the factors causing male infertility [35,41]. In view of concept of the beneficial effect of granulosa cells on oocyte maturation and the need for independent prognostic markers for better outcomes with conventional IVF for couples with non-male factor infertility, we focused on survivin as target genes in luteinized granulosa cells of the human ovaries.

The purpose of this study was to determine whether the incidence of survivin expression in human luteinized follicular granulosa cells shows geographic variations and whether there is any relationship of the expressed gene with infertile clinical features and outcomes after IVF or ICSI and embryo transfer (ET).

## Methods

### Patient sample

29 women who had been subjected to IVF or ICSI and ET were enrolled in the study in order to determine the expression of survivin mRNA in their ovarian follicular granulosa cells. Among them, 19 cases (65.5%) underwent IVF due to tubal disease and 10 cases (34.5%) underwent ICSI due to male infertility. All samples were acquired from patients that visited the 1^st^ Obstetrics – Gynecology Department of “Alexandra” General Hospital, Athens during 2011 for assisted reproduction. The patients were subjected to the same ovulation protocol. Written informed consent was obtained from the participants of this study. Women with history of diabetes mellitus and/or polycystic ovarian syndrome (PCOS), as well as women with endometriosis were excluded from the study.

### Hormone assays

The hormone levels were evaluated using radioimmunoassay (RIA) commercially available kits. Levels of FSH, LH, oestradiol and Antimüllerian hormone (AMH) were determined at the 2^nd^ to 5^th^ day of the menstrual cycle. Serum prolactine (PRL) levels were also determined within one of the six previous menstrual cycles. Also, serum oestradiol levels were measured on the 5^th^ day of rFSH administration and on the day of hCG administration.

### Protocol for controlled ovarian hyperstimulation and follicle monitoring

Commercially available GnRH-analogue (Suprefact, Buserelin; Hoechst, Frankfurt, Germany) was self-administered subcutaneously (sc) into the thigh at a dose of 200 μg/day, starting on the midluteal phase of the preceding menstruation cycle and continuing until 24 h before the administration of hCG. Treatment with rFSH (Gonal-F; Serono, Geneva, Switzerland) was started after 14 days with 225 IU/day and continued until the administration of hCG for ovulation induction. Serum oestradiol and ovarian suppression were evaluated prior to the administration of the exogenous gonadotropins. rFSH dose was administered as a sc injection in the abdomen and readjusted upon response, based on ultrasound and serum E_2_ levels, with a maximum does of 450 IU/day. The dose was reduced or discontinued if the patient was at risk of developing OHSS. Ovulation was induced with 10,000 IU of hCG within 24 h after the last rFSH and GnRH-a administration, preferably when all of the following criteria had been met: the largest follicle had reached a mean diameter of at least 18 mm, 2 or more other follicles had a mean diameter of 16 mm, and serum estradiol (E_2_) levels were within an acceptable range for the number of follicles present. All follicles of >10 mm in diameter were retrieved with follicle aspiration 36 hours after hCG injection by transvaginal ultrasound-guided aspiration. Maturity of oocytes was assessed by microscopic examination. An oocyte found at least in the metaphase II stage (MII) (production of one polar body) was considered as a mature.

In the case of IVF, insemination was withheld with insemination medium 6 hours after oocyte retrieval, and fertilization was confirmed by identification of pronuclei 16 hours after insemination. The participating patients of this group gave written consent some of the cumulus–mature oocyte complexes (CMOCs) to be used only for the study: the cumulus–mature oocyte complexes (CMOCs) were manually denuded separately using a fire-polished tip glass pipette, these granulosa cells were analyzed for each patient separately, but the corresponding mature oocytes were not used for fertilization.

In case of ICSI, the cumulus-mature oocytes complexes (CMOCs) were manually denuded from granulosa cells using a fire-polished tip glass pipette. Granulosa cells from all mature oocytes per patient were collected together. ICSI was performed only in oocytes that were morphologically confirmed to be in metaphase II with the first polar body extruded (mature oocytes). Motile spermatozoa were selected with the swim-up technique, which consists of washing the semen with the Gamete medium and centrifugation at 300 g. The supernatant was discharged and in the same tube 1 ml of the medium was added and left standing for 30 min to allow the sperm to swim up from the pellet. Just before fertilization 2 μL of the sperm suspension were added to 5 μL of Polyvinylpyrrolidone solution (PVP) in order to reduce the spermatozoa mobility. A single spermatozoon was retrieved and injected to the cytoplasm of the oocyte through the zona pellucida. The fertilized oocytes were placed in plates with 1 mL of IVF nutrient and were observed using stereomicroscope 16 to 18 hours later for the presence of pronuclei and polar bodies.

Fertilization was considered normal when the oocytes contained two pronuclei (2PN)/two polar body stage (2PN/2 PB) 16 hours after fertilization. Three embryos were replaced in the uterine cavity on day 2 or 3 after fertilization. Micronized progesterone (P_4_) (Utrogestan, Faran, Greece) (200 mg three times daily) was administered by the vaginal route as luteal phase support, starting after oocyte collection and continued until menses or during the first three weeks of pregnancy. P_4_ treatment was continued up to menstruation or for at least the first 3 weeks if the patient became pregnant. Definition of pregnancy required a positive β-hCG test 14 days after embryo transfer. Definition of a clinical pregnancy required an endometrial gestational sac with a transvaginal ultrasound scan, which continued the development until at least the twelfth gestational week. At the midluteal phase, careful abdominal ultrasound assessment was performed to record any signs of OHSS. The patient then was followed up, and the outcome (clinical pregnancy or menstruation) was recorded.

### Evaluated parameters

For the patients enrolled in the study the following characteristics were recorded: age, body mass index (BMI), duration of infertility, previous assisted reproduction attempts, serum base levels of FSH and LH and Antimüllerian hormone (AMH) levels at the beginning of the menstrual circle, as well as serum prolactine (PRL) in one of the past 6 menstrual cycles. Parameters associated with ovulation stimulation were also recorded: total duration of ovulation stimulation (days), total dose of rFSH used and serum beta-oestradiol levels on the day of hCG administration. Moreover, we kept records of the number of follicles aspirated and the total number of oocytes retrieved. Evaluated parameters associated with oocyte developmental competence included: (i) the number of mature oocytes found in metaphase II (MII), (ii) the oocyte maturation rate, (iii) the rate of fertilization as the ratio of two pronuclei observed 16-18 h after semen administration by the number of intact oocytes after IVF or ICSI (2PN) and the 7-cell stage on day 3, (iv) the degree of fragmentation in embryos, and the ratio of good quality embryos as the ratio of embryos with at least 7 cells and <10 % fragmentation on day 3 after IVF or ICSI, (grade 1 to grade 3). All the above parameters, as well as the outcome of IVF or ICSI (clinical pregnancy, biochemical pregnancy, failure to induce pregnancy) were evaluated in association with survivin mRNA expression.

### RNA isolation and preparation of cDNA from granulosa cells

In order to determine survivin mRNA expression, cumulus cells were collected during oocyte retrieval. The cells were segregated from cumulus-mature oocyte complexes (CMOCs) through the process of stripping using a fire-polished tip glass pipette. RNA was extracted using the RNeasy Micro Kit (Qiagen, Valencia, CA, USA). RNA extraction was conducted according to manufacturer’s protocol. The extracted RNA was a product of cumulus cells pooled from several CMOCs and not only from the oocytes that proceeded to embryo transfer. Moreover, RNA concentration of each sample was determined by spectrophotometry and its quality was evaluated by agarose gel electrophoresis. cDNA preparation was performed using 20 ng aliquots of total RNA extracted. RNA was reverse-transcribed using 0.5 mM dNTP mix (Ambion, Austin, Tx, USA), 5 μM oligo dT Primer (Ambion, Austin, Tx, USA), 1xRT buffer (Ambion, Austin, Tx, USA), 80 U ribonuclease inhibitor (Invitrogen Life Technologies), 1600 U M-MLV reverse transcriptase (Invitrogen Life Technologies) and nuclease free water (Ambion, Austin, Tx, USA) to a total volume of 40 μl. The reactions were carried out in Mastercycler (Eppendorf) with the following conditions: 80°C for 3 min, 42°C for 60 min and 92°C for 10 min. The resulting cDNAs were stored at -20°C.

### Quantitative real time-polymerase chain reaction analysis

The expression of ABL and survivin mRNA in luteinized granulosa cells were assessed by real-time PCR using sense and antisense primer pairs and hybridization probes for the genes of interest as described by Emig M, et al. [42] for ABL, and by Steffen et al. [43] for survivin generating a 338 and 379 base pair products respectively. The primers of each set were intended to bind to different exons to avoid amplification of contaminating genomic DNA and to eliminate mis-priming events generating detectable signal. The specific primers and probes were used at a concentration of 0.5 μl and 0.5 μl in each reaction respectively. To determine the steady amount for survivin mRNA levels in granulosa cells, a quantitative competitive PCR (QC RT-PCRE) was developed using a LightCycler 480 (Roche). All samples were run in duplicate and no template controls were included in all runs to exclude possible DNA contaminations. Reaction volume was 20 μl and carried out with 2x mastermix (QuantiTect Multiplex PCR Master Mix, Qiagen, Berlin, Germany) 10 μl, 10pmol of each 3′ and 5′ primer (TIB MolBiol, Berlin, Germany) 0.5 μl, 5pmol of each probe (TIB MolBiol, Berlin, Germany) 0.25 μl, 2 μl cDNA (from 1 μg RNA) and adjusted to 20 μl reaction final volume with ddH_2_O. Then mixes were incubated in the Light Cycler instrument (Roche Molecular Systems, Alameda, CA). Forty-five cycles of PCR amplification were run with 95°C for 15 s for denaturation, 64°C for annealing 30 s, and 72°C for 20 s for extension. Melting curve experiments had previously established that the fluorescence signal for each amplicon was derived from the products only, and no primers dimmers were found.

### Statistical analyses

All statistical analyses were performed using the SATA 9 statistical software. Differences between qualitative/categorical variables were evaluated with the *x*^2^ of Pearson. Non-parametric Wilcoxon rank-sum and Kruskal-Wallis tests were used to compare differences of quantitative variables between categories of qualitative variables. The Spearman rank correlation coefficient (Spearman’s rho) was used to analyze the relationship between two different values. Multiple linear regression analysis and multiple logistic regression analysis were used for the detection of parameters related with the levels of survivin gene expression. A *p* value <0.05 was considered statistically significant.

## Results

### Patients' characteristics

The average age of the patients was 36.03 ± 4.75 years, their average BMI was 22.86 ± 4.04, the average basal FSH levels (IU/l) were 6.59 ±2.06, and the average prolactin (PRL) levels (ng/ml) were 13.92 ± 6.73. Table 1 presents additional demographic and clinical parameters. No statistically significant differences were found between tubal or male factor infertility and age of women, prolactin levels, estradiol levels on the fifth day of rFSH administration and the number of oocytes retrieved (Table 2).

**Table 1:** Patients’ characteristics

**Characteristics**	**Median (IQR)**
Infertility duration (years)	4.00 (3.00, 6.00)
LH (IU/L)	4.55 (3.37, 5.97)
AMH (μg/L)	5.70 (3.10, 12.20)
Total gonadotropin dose	2,900.00 (2,275, 3,650.00)
Duration of Stimulation	10.00 (9.00, 11.00)
Serum estradiol on the 5^th^ day of rFSH administration (pg/ml)	401.00 (300.00, 585.00)
Serum estradiol on the day of hCG administration (pg/ml)	2,022.00 (1,561.00, 2,569.00)
Number of follicles aspirated	9.00 (7.00, 10.00)
Grade 2 embryos	0.00 (0.00, 2.00)
Grade 3 embryos	4.00 (3.00, 7.00)
High quality embryo ratio (%)	100.00 (66.67, 100.00)

Patients’ demographics and clinical parameters.

**Table 2:** Infertility causes cycle characteristics

**Infertility causes**	**Male factor**	**Tubal factor**	*p*value*
Age (years)	36.50 (33.00, 39.00)	36.00 (33.00, 37.00)	0.564
PRL (ng/ml)	12.7 (7.2, 17.0)	14.70 (9.00, 16.60)	0.920
Estradiol, 5^th^ day of rFSH administration (pg/mL)	321.50 (300.00, 498.00)	450.00 (267.00, 758.00)	0.251
Number of oocytes retrieved	8.50 (6.00, 10.00)	8.00 (6.00, 10.00)	0.693

*Statistical test.

Male or tubal infertility causes in correlation with patients’ cycle characteristics.

Fertilization was successful in all cases in the laboratory (29/29). A pregnancy test was positive in 31% (n = 9) of cases and a clinical pregnancy was confirmed in 21% (n = 6) of women. No multiple pregnancies were observed, while a biochemical pregnancy, a first trimester abortion and an ectopic pregnancy occurred in 1 case each. Positive pregnancy test was found in 4 cases in the ICSI group and in 5 cases in the IVF group. Clinical pregnancy was found in 3 cases in each group. The median for age in the ICSI group was 36.5 (IQR: 33.0-39.0), while in the IVF group was 36.0 (IQR: 33.0-37.0). The median for PRL in the ICSI group was 12.7 (IQR: 7.2-17.0) and in the IVF group was 14.7 (IQR: 9.0-16.6). The median for estradiol levels (pg/ml) the fifth day of rFSH administration in the ICSI group was 321.5 (IQR: 300.0-498.0), while in the IVF group was 450.0 (IQR: 267.0-758.0). The median number of oocytes retrieved in the ICSI group was 8.5 (IQR: 6.0-10.0), while in the IVF group was 8.0 (IQR: 6.0-10.0).

### Incidence of survivin gene expression in granulosa cells

Survivin gene expression in luteinized granulosa cells in women that underwent IVF or ICSI was observed in 93% of the studied cases (27 out of 29) and the median survivin mRNA/ABL mRNA level was 0.45 (quartile range 0.22 – 2.94).

### Expression of survivin gene in granulosa cells according to clinical parameters

**Figure 1:**
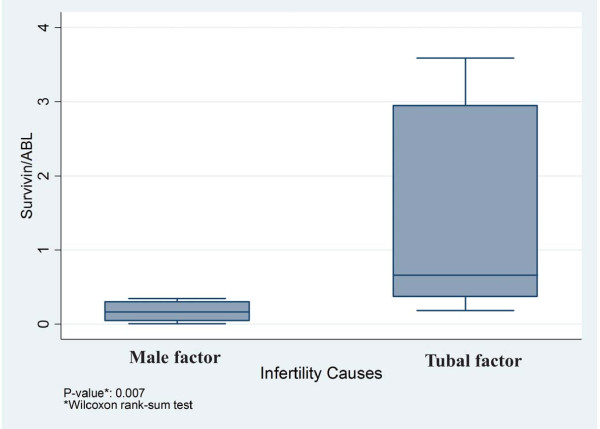
Higher levels of survivin gene expression are shown in cases with tubal factor infertility compared to normal women (male factor infertility) (Wilcoxon rank-sum test, p = 0.007).

Higher levels of survivin gene expression were found in the cases with tubal factor infertility compared to normal women (male factor infertility) (Wilcoxon rank-sum test, p = 0.007) (Figure 1). Also, it is obvious that the same statistical significance was found between IVF and ICSI method of treatment, respectively. There were no significant differences among the levels of survivin gene expression and age, BMI, years and causes of infertility, previous assisted reproduction attempts, basal serum FSH and LH levels, serum levels of PRL and AMH, serum oestradiol levels on the fifth day of rFSH administration and on the day of hCG administration, the total dose of rFSH, the duration of treatment, the number of follicles aspirated, the total number of oocytes retrieved, the number of mature oocytes retrieved, the mature oocytes ratio (<60%, 60-75% and ≥75%), the embryo grade, the positive pregnancy test and the existence of clinical pregnancy.

When embryo grade (grade 3, grade 3 + 2, grade 2) was used as the dependent variable, whereas age (ys), PRL (ng/ml), estradiol levels the fifth day of rFSH administration (pg/ml) and survivin/ABL mRNA expression were used as independent variables, only estradiol levels on the fifth day of rFSH administration showed a trend for correlation to the embryo grade, however it did not reach statistical significance (p = 0.087) (Table 3). Additionally, when the clinical pregnancy was used as dependent variables and the above parameters as independent variables no correlations were found respectively. Only a trend for correlation but not statistically significant was found between clinical pregnancy and age or PRL levels (p = 0.069 and p = 0.054 respectively) (Table 4). Finally, the levels of survivin gene expression were used as dependent variable, whereas age (ys), BMI, peak levels of estradiol the day of hCG administrion, number of oocytes retrieved, mature oocyte ratio and total dose of rFSH administrated were as independent variables, but this analysis yielded no statistically significant results.

**Table 3:** Embryo grade cycle characteristics

**Characteristics**	**Embryo grade**			*p*value*
	**Grade 3**	**Grade 3 + 2**	**Grade 2**	
Age (years)	36.00 (35.00, 37.00)	38.00 (36.00, 41.00)	32.50 (29.50, 35.00)	0.178
FSH (IU/L)	6.00 (4.90, 7.50)	9.40 (5.50, 9.80)	6.30 (6.20, 7.20)	0.402
PRL (ng/mL)	12.10 (9.00, 16.60)	13.90 (4.80, 22.30)	14.90 (9.40, 16.60)	0.974
Total gonadotropin dosage	2,800.00 (2,300.00, 3,400.00)	2,550.00 (1,700.00, 3,075.00)	3,837.50 (2,837.50, 4,025.00)	0.197
Estradiol, 5^th^ day of rFSH administration (pg/mL)	474.00 (306.50, 697.50)	401.00 (300.00, 509.00)	233.00 (110.00, 321.50)	0.087
Estradiol, peak (pg/mL)	2,062.50 (1,630.50, 2,593.00)	1570.00 (1,254.00, 2,795.00)	1,925.00 (1,425.50, 2,034.00)	0.706
Number of oocytes retrieved	9.00 (6.50, 10.00)	7.00 (5.00, 10.00)	8.00 (6.00, 8.50)	0.713
Mature oocyte ratio (%)	67.00 (61.50, 78.00)	80.00 (70.00, 91.00)	75.00 (62.50, 76.50)	0.504
Survivin/ABL	0.60 (0.30, 3.10)	0.20 (0.00, 2.50)	0.20 (0.10, 0.50)	0.153

*Statistical Test.

Embryo grade in correlation with patients’ cycle characteristics and survivin/ABL genes expression.

**Table 4:** Clinical pregnancy cycle characteristics

**Characteristics**	**Clinical pregnancy**		
	**No**	**Yes**	*p*value*
	**Median [IQR]**	**Median [IQR]**	
Age (years)	36.00 (33.00, 37.00)	38.00 (36.00, 44.00)	0.069
FSH (IU/L)	6.3 (5.50, 7.90)	5.5 (4.10, 8.50)	0.634
PRL (ng/mL)	12.10 (7.20, 16.1)	19.20 (11.70, 22.60)	0.054
Total gonadotropin dosage	3,075.00 (2,275.00, 3,675.00)	2,625.00 (2,325.00, 3,175.00)	0.398
Estradiol, 5^th^ day of rFSH administration (pg/mL)	400.00 (252.00, 691.00)	450.00 (309.00, 509.00)	0.572
Estradiol, peak (pg/mL)	2,022.00 (1,561.00, 2,569.00)	2,188.50 (1,254.00, 2,805.00)	0.686
Number of oocytes retrieved	8.00 (6.00, 10.00)	9.00 (6.00, 10.00)	0.765
Mature oocyte ratio (%)	71.00 (60.00, 78.00)	68.50 (67.00, 83.00)	1.000
Survivin/ABL	0.50 (0.20, 3.00)	0.3 (0.20, 1.20)	0.246

*Statistical Test.

Clinical pregnancy in correlation with patients’ cycle characteristics and survivin/ABL genes expression.

## Discussion

Apoptosis is a genetically determined and biologically functional mode of cell death. It plays important roles in numerous physiological events in mammals, including ovarian follicular atresia, decidualisation and placentation during embryo implantation, as well as many pathological conditions [44,45]. The major features of apoptosis are internucleosomal DNA fragmentation, cell shrinkage, plasma membrane blebbing, formation of apoptotic bodies and phagocytosis of apoptotic bodies by macrophages [46]. During follicular growth, more than 99% of follicles disappear, primarily due to apoptosis of granulosa cells. Follicular atresia is a hormonally regulated process, and different factors are affecting the decision to die at different stages of ovarian follicle development [47]. Atretogenic factors include caspases, protein bax, members of the tumor necrosis factor family (TNF-α, Fas, FasL, TRAIL), tumor suppressor protein P53, members of transforming growth factor-beta family (factor NODAL and AMH), c-Myc, endothelins, androgens and GnRH [44]. Successful follicle development depends on the presence of survival factors that promote follicle growth and also protect cells from apoptosis. These include factors produced within the ovary as well as the gonadotropins LH and FSH. Some of the paracrine factors that promote survival during the growth and differentiation of follicles include kinase Akt, members of bcl-2 family, KIT-ligand and c-KIT receptors, stem cell factor (SCF), members of TGF-beta family (activin, factors BMP-4 and BMP-7, growth differention factor-9), oestrogens, insulin and IGFs, epidermal growth factor (EGF), basic fibroblast growth factor (bFGF), TGF-α, interleukin 1b (IL-1b), growth hormone (GH) and the member of inhibitor of apoptosis, survivin [44,47,48]. Most of the inhibitors of follicle atresia are regulated by FSH and LH. When the growing follicles reach the antral stage, they express receptors for FSH and become dependent on FSH stimulation. Sufficient FSH concentrations are critical for survival of follicles that have differentiated to the antral stage or beyond. During each reproductive cycle, increasing FSH concentrations rescue developing follicles. LH is important for follicles approaching ovulation and expressing LH receptor [47]. FSH and LH influence oocyte growth and maturation through the sterol pathways in mice [49]. Follicular fluid meiosis-activating sterol (FF-MAS) is found at high concentrations in the follicular fluid of mammals including humans in response to gonadotropins and is proved to be stimulatory to oocyte meiotic resumption, while lanosterol 14α-demethylase (LDM), a key enzyme that converts lanosterol to FF-MAS seems to have a positive effect on the oocyte plasma maturation for fertilization and early embryo development in mice [50]. In addition, epidermal growth factor receptor (EGFR) activation, by protein kinase C (PKC) signal pathway, participates in FSH-induced oocyte maturation in pigs [51]. It is well known that the expression of the LH receptor (LHR) in cumulus cells is related with FSH-induced meiotic resumption of cumulus enclosed oocytes (CEOs). An important new step towards understanding the physiological actions of gonadotropins during oocyte maturation is the finding that in mice the LHR expression in cumulus cells has a functional role during FSH-induced oocyte maturation, which process is possibly regulated by MAPK cascade [52]. In addition to all that it has been found that in mice FSH increases cAMP-dependent protein kinase (PKA) levels and induces cAMP response element-binding protein (CREB) phosphorylation and cytochrome P_450_ lanosterol 14α-demethylase (CYP51) expression in cumulus cells before the oocyte meiotic resumption [53]. In the absence of survival factors, endogenous apoptosis pathways within the follicle become activated and lead to follicular atresia [47]. The present study showed the expression of survivin in luteinized granulosa cells from a sample of Greek patients that underwent IVF or ICSI. To the best of our knowledge, this is the first report which determined the frequency of survivin expression in these cells from a sample of Greek patients. Therefore, the present study gives an additional estimation on the frequency of expression of the survivin in luteinized granulosa cells, which is detected in almost all of the studied cases from Greek patients. However, more studies are needed to analyze larger numbers from Greek patients and investigate therefore whether geographic or ethnic prevalence of survivin gene expression could be caused by environmental or genetic factors. The expression of survivin, in granulosa cells reported herein, is in agreement with the previous findings from Fujino et al. [34], who studied the expression of survivin gene expression in granulosa cells from infertile Japanese patients and found such expression in all granulosa cells. In addition, the present study found a statistically significant increased expression of survivin in granulosa cells of women who had tubal factor infertility compared with normal women (male factor infertility). This finding suggests a protective role of survivin in the ovarian microenvironment from situations such as inflammation (salpingitis), hydrosalpinx, tubal ligation and salpingectomy. Ovrieto et al., (2011) studied the effects of salpingectomy due to hydrosalpinx on the outcomes of assisted reproduction and embryo transfer and found a significant reduction in ipsilateral ovarian response to ovulation induction as regards the development of follicles [54]. It is therefore likely that survivin might try to protect the ovaries, with possible influenced perfusion due to the ipsilateral salpingectomy. In cases with tubal inflammation or hydrosalpinges survivin might try to protect the ovaries from follicular apoptosis in a paracrine environment. However, more studies are needed to support our hypothesis probably using animal models.

In the present study the capacity for fertilization (fertilizability) was 100%, regardless IVF or ICSI method used. An increased survivin expression in granulosa cells was found in women undergoing IVF compared with ICSI. Nakahara et al., (1997) suggested that when the quality of eggs is small, measured by apoptosis in granulosa cells, then the eggs are more likely to be fertilized by ICSI compared with IVF method [11]. However, Clavero et al., (2003) found that the rate of apoptosis in granulosa cells was not associated with the maturity of the oocytes and the ability for fertilization in ICSI or the quality of follicles during the ovulation induction [55]. Despite the controversy that exists in the literature about the effects of apoptosis in luteinized granulosa cells as predictor of oocytes quality during ovulation induction in cycles of IVF or ICSI, the estimation of survival factors in these cells might have prognostic role. An example supporting the validity of using survival factors for prognosis provides the IGFs family. It has been found that the IGF1, IGF2 and their receptors are down regulated in ovarian granulosa cells of women with diminished ovarian reserve (DOR) compared to those with normal ovarian reserve (NOR) undergoing in vitro fertilization (IVF) [56]. However, more research is needed about the clinical significance of the expression of some other survival factors in the luteinized granulosa cells. Data generated using mice genetic models perturbing ovulation and fertility indicates that the EGF-like growth factors amphiregulin, epiregulin, and betacellulin are induced by LH and activate the EGF receptor pathway in granulosa cells of preovulatory follicles to impact ovulation [57,58]. Whether this network also plays a critical role in humans and whether it can be used as a biological marker of follicle development or for the improvement of fertility remains to be determined [57]. We enounce our hypothesis about the levels of survivin mRNA expression in ovarian granulosa cells in tubal factor infertility. Some patients in the subpopulation of women with tubal factor undergoing assisted reproduction and embryo transfer probably could benefit in assessing oocyte quality by measuring the levels of survivin expression in their granulosa cells. Therefore, if the survivin levels in granulosa cells are low, then ICSI should be concerned, as ICSI is an invasive method and good oocyte quality is not required. On the other hand, if survivin levels are highly expressed in granulosa cells then IVF should be preferred, as IVF is a non-invasive method and therefore normal sperm-egg interaction and good oocyte quality is essential. However, the cut-off survivin mRNA expressed levels have to be determined in such cases. Fujino et al., (2008) found that there was a statistically significant correlation in the levels of survivin expression in granulosa cells among women with endometriosis and normal women. In women with endometriosis lower survivin levels were found [34]. However, one factor to be considered is that in the present study only normal women (male factor infertility) and women with tubal factor infertility were studied. Women with endometriosis were not included since endometriosis promotes apoptosis [11]. Also, in the present study women with polycystic ovarian syndrome were not included because androgens promote apoptosis. Fujino at el, (2008) found no statistically significant difference in levels of survivin expression between normal women (male factor infertility) and women with tubal factor infertility [34], as we found in this study. Fujino et al., (2008) found that the survivin expression in granulosa cells was higher in all pregnant women than in not pregnant women [34]. However, this did not result from the findings of this study. As there is some controversy in this field more studies should be taken. In addition, it would be interest further studies to investigate expression with any clinical significance of survivin gene in granulosa cells of patients with diminished ovarian reserve (DOR) as such population was not included in our study.

## Conclusions

There is an expression of survivin gene in luteinized granulosa cells at a ratio of 93% in cases from Greek patients. Higher levels of survivin mRNA expression in luteinized granulosa cells in cases with tubal infertility compared to normal women seem to protect ovaries from follicular apoptosis in a paracrine environment in cases with tubal inflammation or hydrosalpinges or in a reduced ovarian perfusion environment in cases with ipsilateral salpingectomies. It seems that a subpopulation of patients with lower levels of survivin mRNA in granulosa cells during the assisted reproduction treatment might benefit with ICSI, which is an invasive method and therefore no good oocyte quality is required.

## Abbreviations

IVF: In Vitro Fertilization; ICSI: Intra Cytoplasmic Sperm Injection; ET: Embryo Transfer; FSH: Follicle-Stimulating Hormone; rFSH: recombinant FSH; LH: Luteinizing Hormone; LHR: LH Receptor; GnRH: Gonadotropin Releasing Hormone; GnRH-a: GnRH-analogue; ART: Assisted Reproductive Technology; RRM: RNA Recognition Motif; RIA: Radio Immuno Assay; PRL: Prolactine; AMH: AntiMüllerian Hormone; P_4_: Micronized Progesterone; OHSS: Ovarian Hyperstimulation Syndrome; BMI: Body Mass Index; NOR: Normal Ovarian Reserve; DOR: Diminished Ovarian Reserve; FF-MAS: Follicular Fluid Meiosis-Activating Sterol; LDM: Lanosterol 14α-Demethylase; EGFR: Epidermal Growth Factor Receptor; PKC: Protein kinase C; CEOs: Cumulus Enclosed Oocytes; CMOCs: Cumulus-Mature Oocyte Complexes; CREB: CAMP Response Element-Binding protein; CYP51: Cytochrome P_450_ Lanosterol 14αDemethylase.

## Competing interests

The authors declare that they have no competing interests.

## Authors' contributions

MV and KS were responsible for the original conception and design, edition of the manuscript, supervision of the whole attempt, data analysis and interpretation of the results. MM was also responsible for the interpretation of the results. MV was responsible as well for correction, revision, and approval of the final version. KS and AA presided as well over the clinical aspect of the study. KP and MM developed the QC RT-PCR system and performed all QC RT-PCR. MV, KS, KP, MM, ZP, CA and AA were involved in the drafting of the manuscript. All authors read and approved the final manuscript.
